# Revolutionizing head and neck squamous cell carcinoma treatment with nanomedicine in the era of immunotherapy

**DOI:** 10.3389/fimmu.2024.1453753

**Published:** 2024-11-29

**Authors:** Hong-Xia Li, Yu-Wen Gong, Pi-Jun Yan, Yong Xu, Gang Qin, Wei-Ping Wen, Fang-Yuan Teng

**Affiliations:** ^1^ Department of Otolaryngology Head and Neck Surgery, The Affiliated Hospital of Southwest Medical University, Luzhou, Sichuan, China; ^2^ Department of Otolaryngology, Biomedical Innovation Center, The Sixth Affiliated Hospital, Sun Yat-sen University, Guangzhou, Guangdong, China; ^3^ Metabolic Vascular Diseases Key Laboratory of Sichuan Province, Metabolic Vascular Diseases Key Laboratory of Sichuan-Chongqing Cooperation, Department of Endocrinology and Metabolism, Luzhou, Sichuan, China; ^4^ Department of Otolaryngology, The First Affiliated Hospital, Sun Yat-sen University, Guangzhou, Guangdong, China

**Keywords:** head and neck squamous cell carcinoma, nanomedicine, treatment, tumor microenvironment, drug delivery

## Abstract

Head and neck squamous cell carcinoma (HNSCC) is a prevalent malignant tumor globally. Despite advancements in treatment methods, the overall survival rate remains low due to limitations such as poor targeting and low bioavailability, which result in the limited efficacy of traditional drug therapies. Nanomedicine is considered to be a promising strategy in tumor therapy, offering the potential for maximal anti-tumor effects. Nanocarriers can overcome biological barriers, enhance drug delivery efficiency to targeted sites, and minimize damage to normal tissues. Currently, various nano-carriers for drug delivery have been developed to construct new nanomedicine. This review aims to provide an overview of the current status of HNSCC treatment and the necessity of nanomedicine in improving treatment outcomes. Moreover, it delves into the research progress of nanomedicine in HNSCC treatment, with a focus on enhancing radiation sensitivity, improving the efficacy of tumor immunotherapy, effectively delivering chemotherapy drugs, and utilizing small molecule inhibitors. Finally, this article discussed the challenges and prospects of applying nanomedicine in cancer treatment.

## 1 Introduction

**Figure 1:**
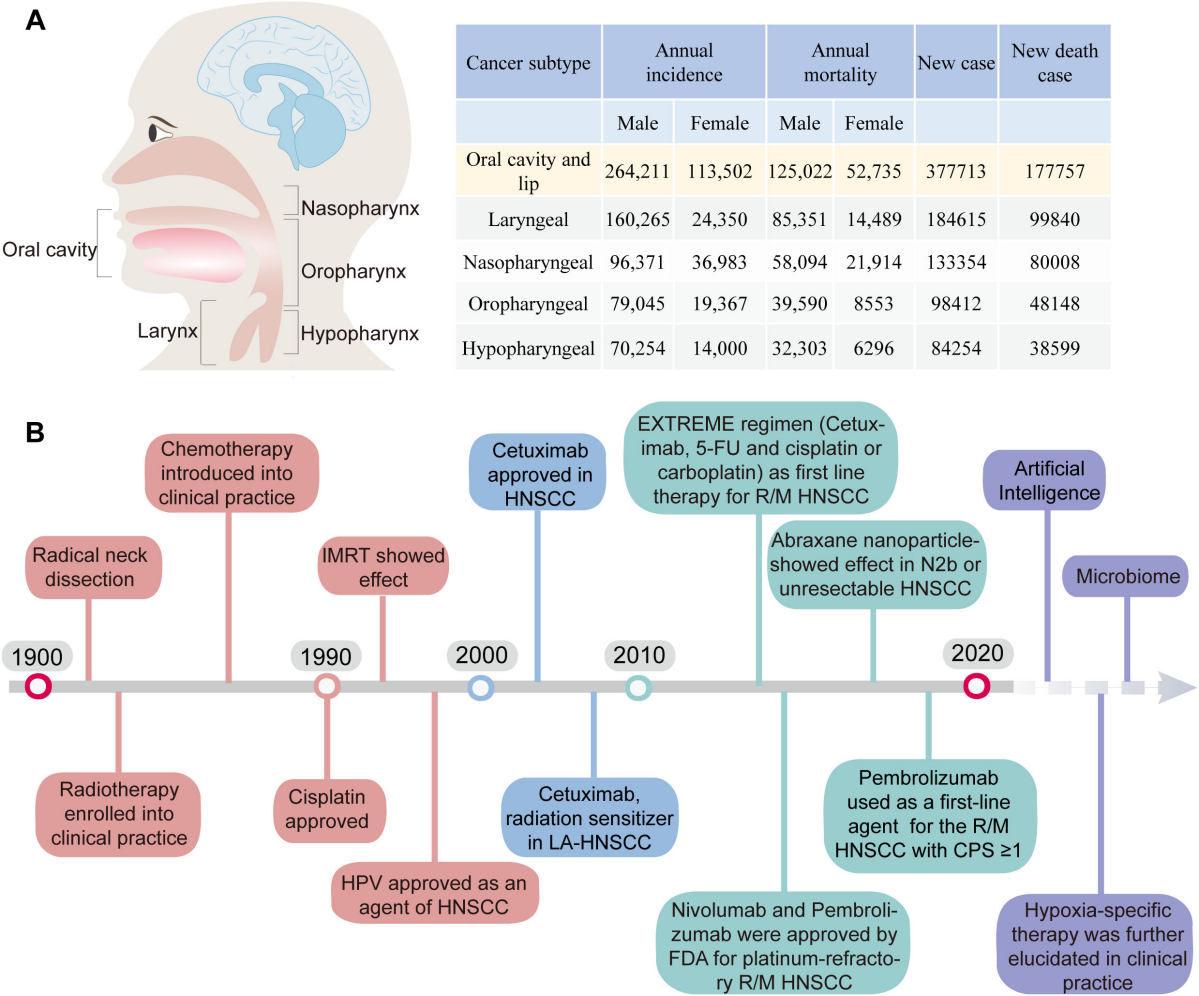
**(A)** The incidence and mortality rates of HNSCC. **(B)** Timeline of the Development of Treatment Regimens and Targeted Therapy for HNC and Future Exploration (1).

Head and neck squamous cell carcinoma (HNSCC) is the predominant malignant neoplasm in the head and neck region, originating from the mucosal epithelium of the oral cavity, pharynx, and larynx. Based on data from the GLOBOCAN, in 2020, there were approximately 930,000 new cases of HNSCC documented globally, leading to 467,000 mortalities. Regretfully, the incidence and mortality of HNSCC have persistently escalated (1, 2) (**Figure 1A**). Given the complex and obscured anatomy of the head and neck region coupled with the paucity of initial symptoms, the majority of HNSCC patients are diagnosed with advanced-stage malignancies when deemed necessary for medical attention. Furthermore, the precise location of head and neck cancer (HNC) can profoundly affect essential sensory functions, leading to impaired emotional health and restricted social functioning. Unfortunately, A comprehensive analysis of HNSCC cases from the Surveillance, Epidemiology, and End Results database, demonstrated that the 5-year and 10-year overall survival (OS) rates for HNSCC were 46% and 31%, respectively (3). Despite extensive improvements in HNSCC treatment, upwards of 65% of patients encounter recurrence or metastasis (4). For locally recurrent malignancies that fail to respond to rescue surgery, radiation therapy, or a combination of both, the prognosis becomes exceedingly dire, akin to distant metastases. Without intervention, survival is confined to only 6-9 months, underscoring the ongoing difficulty of attaining long-term remission and disease control (5).

HPV vaccines have the potential to serve as an effective preventive measure against HPV^+^ HNSCC (6, 7). Whereas HPV^-^ HNSCC are more heterogeneous in outcome (8). Surgery or radiation therapy can serve as feasible options for early-stage oral and laryngeal malignancies (9). Nevertheless, approximately 70%-80% of HNSCC patients presenting with advanced stage III/IV tumors often experience disease progression associated with extensive local invasion and regional lymph node dissemination. Contemporary treatment strategies for advanced HNSCC encompass surgery, radiotherapy, and systemic therapy (**Figure 1B**).

In 2006, the FDA approved cetuximab, an EGFR inhibitor, as a first-line treatment for recurrent or metastatic (R/M) HNSCC. Cetuximab has shown significant effectiveness as a radiosensitizer, either alone or in combination with chemotherapy for R/M HNSCC (10). It has unequivocally demonstrated that cetuximab exhibits the ability to downregulate IFN-γ and induce the expression of programmed death ligand 1 (PD-L1) in HNSCC (11). Nevertheless, cetuximab therapy may potentially augment the influx of immunoregulatory regulatory T cells (Tregs) within the tumor microenvironment (TME) (12). Additionally, it may hinder the cytotoxic abilities of activated CD8^+^ T cells, as indicated by the increased co-expression of programmed death receptor 1 (PD-1) and T-cell immunoglobulin and mucin domain 3 (13). These findings highlight the dual influence of cetuximab therapy.

The emergence of immune checkpoint inhibitors (ICIs) has brought about a revolutionary shift in cancer treatment. Notably, between 2015 and 2019, there was an impressive 8.2% reduction in mortality rates (14). At present, the ICIs have consistently exhibited significant therapeutic effects in melanoma, non-small cell lung cancer (NSCLC), glioblastoma, and advanced skin squamous cell carcinoma (15–20). Pembrolizumab and nivolumab were FDA-approved for the treatment of R/M HNSCC in 2016 and 2019 (21–23). Up to September 30th, 2024, the FDA has approved 196 clinical trials investigating immunotherapy for HNSCC (2, 24, 25). Of these, 74 are currently in active participant recruitment, 32 have been initiated but not yet recruited, and 37 have concluded. However, completed trials have shown a relatively low durable response rate of 15%-20% among patients (26). Furthermore, some patients may experience significant immune-related side effects, such as dermatitis, colitis, hepatitis, and pneumonitis, which may require temporary suspension of ICI treatment or alternative interventions (27, 28).

In recent years, nanomedicine has undergone substantial advancement in oncological treatment (29). It proffers several advantages to cancer therapy, encompassing augmented drug availability, diminished side effects, and precise drug delivery (30–33). A variety of nanoparticle (NP) categories have been leveraged for tumor diagnostics and treatments (34–36). This review provides a synopsis of nanomedicine research progression in HNSCC treatment, emphasizing the extraordinary advancements in nanomedicine-based immunotherapeutic strategies.

## 2 The advantage of nanomedicines application in HNSCC

Although HNSCC is a solid tumor, due to its special anatomical location, nanomedicines exhibit greater specialized benefits in addressing HNSCC compared with other solid tumors. Primarily, radiotherapy serves as a prevalent treatment option for HNSCC. Consequently, nanomedicine holds potential application possibilities in treating HNSCC via leveraging its radiosensitizing effect. Additionally, the cervical region is abundant in lymphatic tissue, thereby rendering HNSCC susceptible to lymph node (LN) metastasis, resulting in recurrence and metastasis. Through modifications of nanomedicine, the LN targeting of nanomedicine can mitigate tumor LN metastasis and enhance patient survival rates. For instance, Liang et al. engineered a hybrid nano-vaccine (Hy-M-Exo) by amalgamating tumor-derived exosomes (TEX) and dendritic cell membrane vesicles (DCMV), which inherited CCR7, a pivotal lymphatic homing protein of DCMV, and enhanced targeting efficacy against LN. Concurrently, Hy-M-Exo stimulated retained tumor antigens and endogenous danger signals in antigen-presenting cells (APCs), inciting a robust T-cell response (37).

NPs are artificially manufactured particles ranging from 1 to 100 nm (38). They come in various types, including metal NPs, metal oxide NPs, mesoporous NPs, polymer NPs, micelles, liposomes, and others (39). These NPs have special characteristics like large surface area, strong electrical conductivity, optical absorption spectral shift, and unique fluorescence properties. Nanomaterial delivery systems offer several advantages in HNSCC treatment, making them a promising therapeutic approach. These advantages encompass refined targeting, precise drug delivery, multifunctional carriers, enhanced drug stability, photothermal therapy augmentation, and solubility/biocompatibility. These features enable nanomaterials to selectively target HNSCC cells, regulate drug release, deliver multiple therapies, safeguard drugs, facilitate photothermal therapy, and demonstrate favorable solubility and biocompatibility.

**Figure 2:**
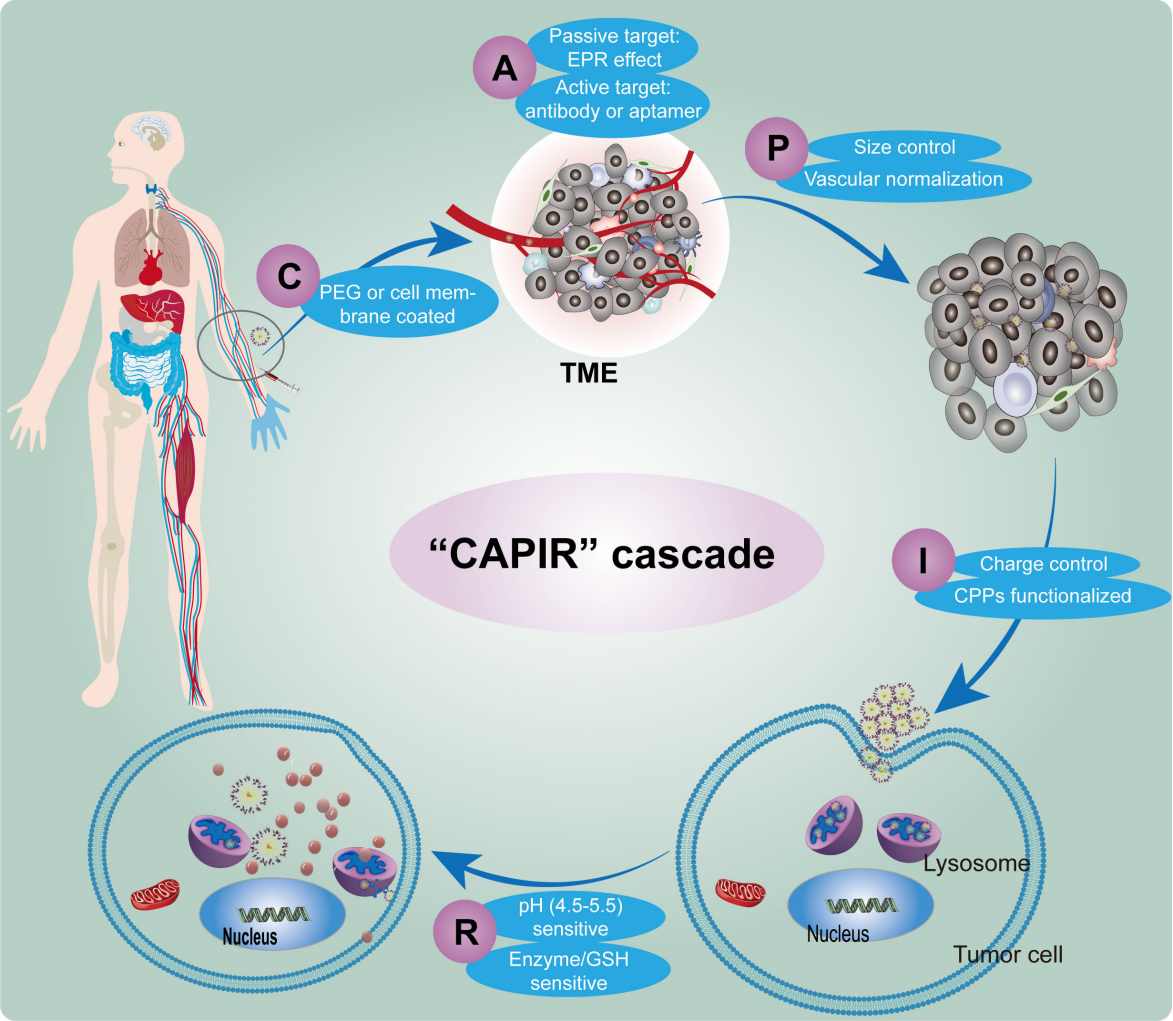
Schematic diagram of the CAPIR cascade of nanomedicine delivering drugs to tumor cell. (C) Circulation within the blood vessels, (A) Accumulation at the tumor site, (P) Penetration into the deeper regions of the tumor tissue, (I) Internalization by tumor cells, (R) Release of therapeutic agents within the intracellular compartment.

Otherwise, the nano-delivery system enters the bloodstream, it undergoes a series of five cascading steps, collectively known as “CAPIR” to achieve successful and efficient delivery of therapeutic drugs to tumor cells. These steps include Circulation within the blood vessels (C), Accumulation at the tumor site (A), Penetration into the deeper regions of the tumor tissue (P), Internalization by tumor cells (I), and Release of therapeutic agents within the intracellular compartment (R) (40). Each step is crucially designed and meticulously constructed to ensure the targeted delivery of nanomedicine to the tumor site (**Figure 2**).

A high-throughput nanoparticle assay was employed to assess the delivery of nucleic acids to HNSCC solid tumors *in vivo* using 94 chemically distinct nanoparticles. DNA barcoding was utilized to identify LNP^HNSCC^, which demonstrated the ability to inhibit HNSCC tropism while minimizing off-target delivery to the liver (41). Meanwhile, NPs possess the capability to modulate various key elements, thereby disrupting the tumor’s immunosuppressive network (42). The dedicated pursuit of NP-based drug delivery holds immense promise in revolutionizing cancer therapy and fostering improved patient outcomes in the foreseeable future. A variety of nanomedicine has been used for HNSCC treatment and summarized in **Table 1**.

**Table 1:** Summary of nano-materials in the therapy of HNSCC.

Nanocarriers	Drug	Effect	Application	Reference
Metal and Metal Oxide Nanoparticles	–	Enhance radiosensitivity	review	(43)
Gold nanoparticles	–	Enhance radiosensitivity	*in vivo* and *in vitro*	(44)
Gold nanoparticles	–	Enhance radiosensitivity	*in vivo* and *in vitro*	(45)
Gold nanoparticles	–	Enhance radiosensitivity	*in vivo* and *in vitro*	(46)
PEG-coated Au-Ag alloy nanoparticles	–	Enhance radiosensitivity	*in vitro*	(47)
High-density lipoprotein nanoparticle	HMG-CoA reductase inhibitors (statins)	Enhance radiosensitivity	*in vivo* and *in vitro*	(48)
Biodegradable gellan- and lipid-based dual nanocarriers	cisplatin and paclitaxel	Enhance radiosensitivity Enhance radiosensitivity	*in vivo* and *in vitro*	(49)
Gadolinium-based nanoparticles	–	Enhance radiosensitivity	*in vitro*	(50)
Tumor antigen-targeted nanosatellite vehicle	SOX2, a novel inhibitor of STING	Nanovaccines for HNSCC	*in vivo* and *in vitro*	(51)
Mesoporous silica rod (MSR)	HPV-16 antigens	Nanovaccines for HNSCC	*in vivo* and *in vitro*	(52)
Ribonucleic acid lipoplex	HPV16 vaccine	Nanovaccines for HNSCC	*in vivo* and *in vitro*	(53)
Injectable nanocomposite hydrogel	–	Nanomedicines for HNSCC by Polarizing TAMs	*in vivo* and *in vitro*	(54)
Injectable nanocomposite hydrogel	a peptide-based proteolysis-targeting chimera (PROTAC) for BMI1 and paclitaxel	Nanomedicines for HNSCC by Increasing TILs	*in vivo* and *in vitro*	(54)
All-trans retinoic acid (ATRA)-poly(lactide-co-glycolide acid) (PLGA)-poly(ethylene glycol) (PEG)	programmed death-ligand 1 (PD-L1)	Nanomedicines for HNSCC by Increasing TILs	*in vivo* and *in vitro*	(55)
Molybdenum disulfide	CpG	Nanomedicines for HNSCC by Increasing TILs	*in vivo* and *in vitro*	(56)
Adhesive hydrogel incorporating silver nanoparticles	bacterium P. anaerobius	Nanomedicines for HNSCC by Increasing TILs	*in vivo* and *in vitro*	(57)
Polymeric micelles	5,10,15,20-tetrakis(meso-hydroxyphenyl)porphyrin (mTHPP)	Nanomedicines for HNSCC by Inducing ICD	*in vitro*	(58)
Zinc oxide nanoparticles	–	Nanomedicines for HNSCC by Inducing ICD	*in vitro*	(59)
Gelatinase-sensitive nanoparticles	photosensitizer and STAT3 inhibitor	Nanomedicines for HNSCC by Inducing ICD	*in vivo* and *in vitro*	(60)
Ruthenium-based photosensitizer (Ru) modified-TiO2 nanoparticles (NPs)	siRNA of hypoxia-inducible factor-1α (HIF-1α)	Nanomedicines for HNSCC by Inducing ICD	*in vivo* and *in vitro*	(61)
Supramolecular Ce6-erastin nanodrug	photosensitizer chlorin e6 (Ce6) and the ferroptosis inducer erastin	Nanomedicines for HNSCC by Inducing ICD	*in vivo* and *in vitro*	(62)
Ligand-decorated PLGA-PEG/NR7 nanoparticles	CDDP	Other applications of nanomedicines	*in vitro*	(63)
CTSB-sensitive amphiphilic polymer	Saracatinib	Other applications of nanomedicines	*in vivo* and *in vitro*	(64)
Cathepsin B (CTSB)-sensitive polymeric drug carrier	Src inhibitor saracatinib (AZD0530) and AKT inhibitor capivasertib (AZD5363)	Other applications of nanomedicines	*in vivo* and *in vitro*	(65)
Polymeric nanoparticles based on α-tocopheryl succinate	PHT-427	Other applications of nanomedicines	*in vitro*	(66)

## 3 Advancements in nanomedicines for HNSCC treatment

### 3.1 Nanomedicines enhance radiosensitivity

Over the past few decades, personalized radiotherapy has made remarkable advancements. However, the occurrence of toxic effects frequently impedes the ability to escalate radiation dosage, thus hindering further improvements in treatment outcomes (67). Radioresistance leads to local control failure in about 40% of HNSCC (68). Enhancing the sensitivity of tumor cells to radiation and achieving tumor eradication with a reduced radiation dose hold paramount importance in HNSCC treatment (69). Brown et al. have identified pivotal factors that influence the efficacy of tumor radiotherapy, encompassing: (1) repair of sublethal cell damage; (2) cell regeneration following irradiation; (3) cell redistribution during the cell cycle; (4) reoxygenation of surviving cells; and (5) intrinsic radiosensitivity (70).

**Figure 3:**
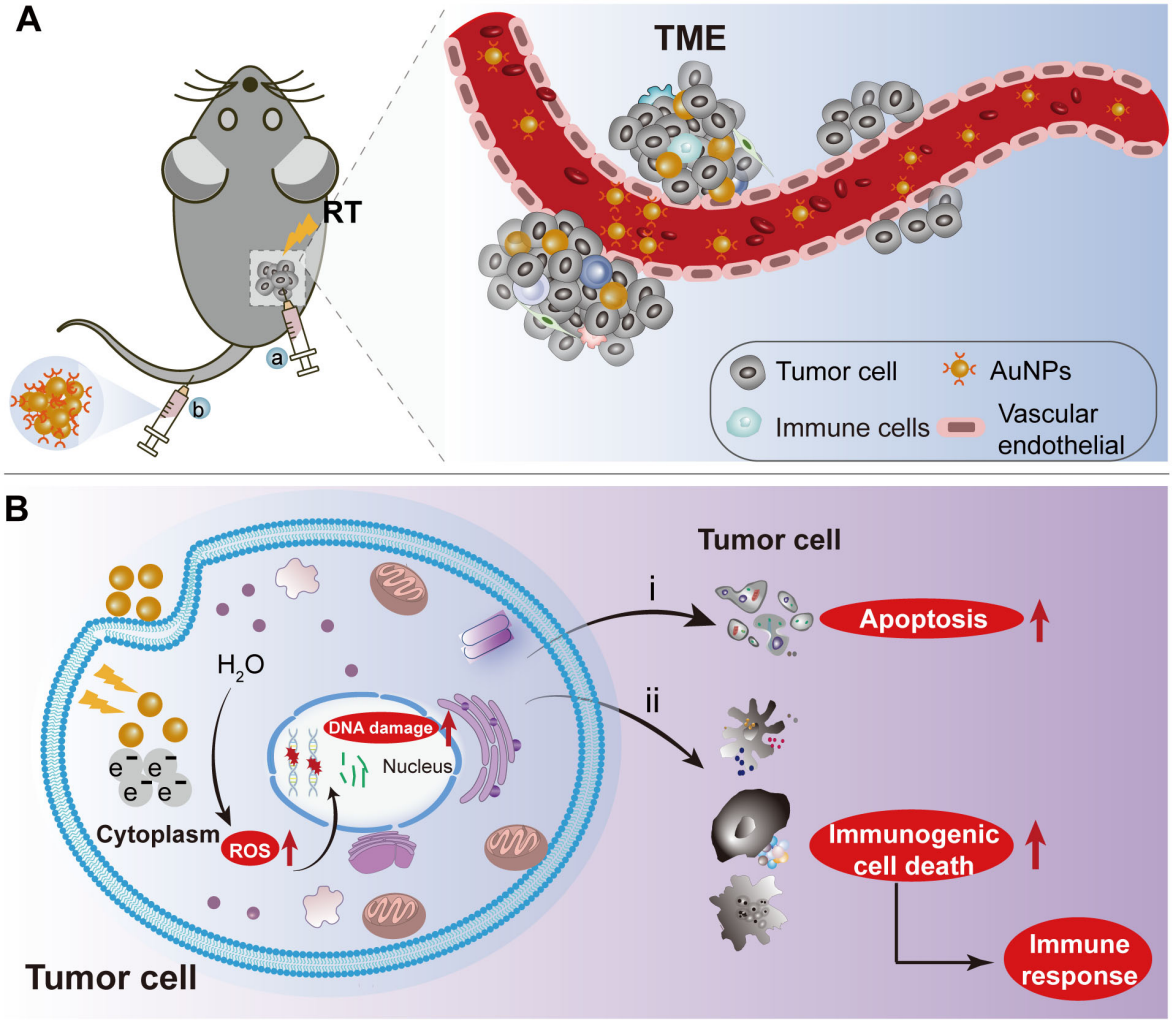
The schematic depiction of AuNPs as radiation sensitizers for HNSCC treatment. **(A)** Nanoparticles were delivered into the tumor microenvironment by intravenous or intratumoral injection of AuNPs combined with RT to tumor-bearing mice. **(B)** The internalization of AuNPs into tumor cells enhances radiation-induced DNA damage and reactive oxygen species (ROS) generation, exacerbating tumor cell apoptosis and necrosis during radiotherapy. Additionally, the localized radiation-induced immunogenic cell death elicits a robust immune response. RT: Radiation therapy. AuNPs: gold nanoparticles.

In the realm of cancer treatment, nanomaterials containing metals such as gold (Au) and silver (Ag) NPs have demonstrated remarkable capabilities in absorbing, scattering, and emitting radiation energy. These NPs can be readily metabolized and eliminated, rendering them a promising avenue for radiosensitization in cancer therapy (71, 72) (**Figure 3**). As an example, Zhang’s team has pioneered the development of gold nanoparticles (GNPs)-based system named Au@MC38, which demonstrates the capability to significantly enhance radiation-induced DNA damage and reactive oxygen species (ROS) production when used in conjunction with radiotherapy. This synergistic approach leads to heightened tumor cell apoptosis and necrosis (73). Additionally, an acid-responsive nano-aggregation system incorporating amycin (Dox) has shown notable radiosensitization effects in esophageal cancer (74).

Remarkable advancements have been achieved in harnessing nanomedicines to augment radiosensitivity in HNSCC (43). Regular administration of GNPs through bi-weekly injections has been observed to have a significant therapeutic effect. This approach demonstrates the immense potential of GNPs in enhancing radiation sensitivity and improving the efficacy of radiotherapy in HNSCC (44–46, 75). Nayak et al. developed polyethylene glycol (PEG)-coated gold-silver alloy nanoparticles (BNPs) that show efficient uptake by oral cancer cells. These BNPs exhibit a radiosensitization ratio of 1.5-1.7, demonstrating a robust radiosensitization effect (47). As previously mentioned, cetuximab is known as a radiosensitizing agent. One effective strategy to overcome tumor radioresistance is to use cetuximab-targeted GNPs to increase the absorption of radiation by tumors at clinically relevant energy and radiation doses (68).

In addition to GNPs, mesoporous silica, liposomes, bovine serum albumin, and polymers, have been extensively investigated as carriers to enhance the effectiveness of radiotherapy (76–81). HMG-CoA reductase inhibitors, commonly referred to as statins, have demonstrated potential anti-tumor and radiosensitizing effects (82–84). Nevertheless, the limited bioavailability of orally administered statins poses a significant challenge. Thierry et al. developed high-density lipoprotein NPs for parenteral delivery of statins. This innovative formulation not only enhances the radiobiological response but also exhibits immunoreactive properties in 2D/3D models of HNSCC (48). Biodegradable gelling-and-lipid dual nanocarrier ion-triggered porous adhesive hydrogels have been used to optimize the targeted delivery of clinical radiosensitizers. This advanced nanosystem facilitates the efficient intracellular uptake and cellular retention of radiosensitizers, resulting in a synergistic enhancement of radiation-induced DNA damage and apoptosis. Furthermore, in-gel NPs have demonstrated improved *in vivo* efficacy of both chemotherapeutic agents by prolonging tumor bioaccumulation and reducing systemic absorption, thereby outperforming systemic commercial agents approved for HNSCC chemoradiotherapy (49). Gadolinium-based NPs (GBNs) have emerged as a highly promising avenue for radiosensitization. Notably, Ardail’s team has achieved remarkable advancements by introducing a novel formulation of GBNs known as AGuIX^®^. These NPs exhibit the ability to accumulate within lysosomes upon cellular uptake, enabling effective radiosensitization specifically for HNSCC (50).

### 3.2 Application of nanomedicines in HNSCC immunotherapy

#### 3.2.1 TME and immunotherapy for HNSCC

**Figure 4:**
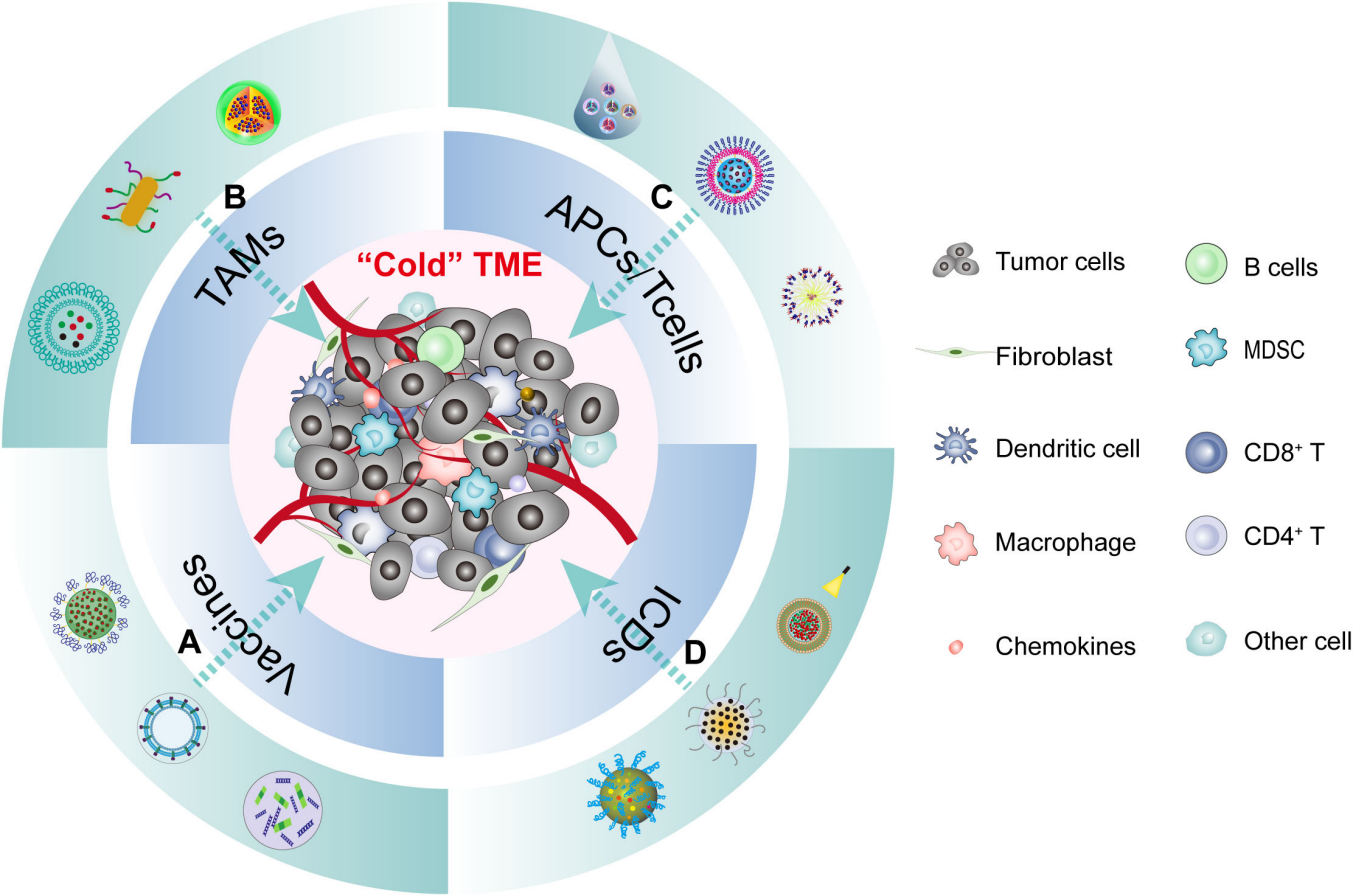
The TME of HNSCC and multifunction of nanomedicine immunotherapy. The cold tumor microenvironment of HNSCC can be reshaped into a hot tumor through various forms of immune microenvironment modulation using different nanoparticles. This process encompasses four main aspects: **(A)** Nanovaccines regulate the TME by triggering innate immunity. **(B)** Nanomedicines polarize TAMs into immunocompetent M1 TAMs. **(C)** Nanomedicines activate tumor-specific immune responses by increasing T-cell infiltration in the TME. **(D)** Nanomedicines stimulate anti-tumor immune responses by inducing ICD in tumor cells.

The TME is an intricate biological system comprising multifaceted components, including tumor cells, tumor-associated fibroblasts, immune cells (including T lymphocytes, tumor-associated macrophages, myeloid-derived suppressor cells, natural killer cells, and tumor-associated neutrophils), and extracellular matrix. The TME serves a pivotal role in the development, progression, and prognosis of tumors (**Figure 4**) (85, 86). In accordance with Daniel’s classification scheme, the TME may be stratified into three phenotypes: the inflamed phenotype, the immune-excluded phenotype, and the immune-desert phenotype (87). In a research conducted by Jakob et al., detailed histological sections of 965 solid tumors were critically assessed to categorize tumor types based on the distribution of immune cells within the TME. The results indicate that in HNSCC, over 50% of tumors displayed infiltration by immunosuppressive Foxp3^+^ Tregs. Moreover, HNSCC frequently demonstrated diminished immune cell populations, including tumors with exclusive lymphocytic or myeloid infiltration (88). Huang et al. executed a proteogenomic analysis of 108 cases of HPV-positive HNSCC and discovered significant deletions in immunomodulatory genes, leading to a low immune infiltration state within the tumors (89, 90). Consequently, the TME of HNSCC can be classified as harboring an immuno-excluded phenotype or an immuno-desert phenotype.

A variety of inflammatory factors and immune cells are present within the TME. Furthermore, the tumor can stimulate an immune response, thereby complicating the complex interplay between the tumor and the immune system (91). Nevertheless, solid tumors can reshape the TME by altering the phenotype of immune cells. Such reshaping encompasses modifications in the expression of immune checkpoint proteins, suppression of effector T cell functionality, and promotion of the transition of pro-inflammatory (M1) tumor-associated macrophages (TAMs) towards anti-inflammatory (M2) TAMs (92). The effectiveness of immunotherapy is intertwined with the immune status of the TME (93). The immune system undergoes dynamic alterations throughout the inception and progression of tumors, as well as subsequent treatments such as chemotherapy, radiotherapy, or immunotherapy (94). In the context of HNSCC, the TME displays considerable heterogeneity and employs diverse mechanisms to evade immune surveillance.

The application of noninvasive nanoparticle-based imaging to visualize TAMs can yield valuable insights into the immune cell composition within the TME (95, 96). This data possesses considerable potential as a guiding principle for immunotherapy. Furthermore, by harnessing nanomaterials for precise and targeted delivery of therapeutic agents, nanomedicine offers an opportunity to enhance the efficacy of immunotherapy whilst simultaneously mitigating treatment-associated side effects (97, 98) (**Figure 4**). This review will explore four facets of nanomedicine in HNSCC immunotherapy (1): Nanovaccines regulate the TME by triggering innate immunity; (2) Nanomedicines polarize TAMs into immunocompetent M1 TAMs; (3) Nanomedicines activate tumor-specific immune responses by augmenting T-cell infiltration in the TME; (4) Nanomedicines stimulate anti-tumor immune responses by inducing ICD.

#### 3.2.2 Nanovaccines for HNSCC

Tumor vaccines are an active form of immune treatment that is garnering significant attention due to their favorable safety profile and minimal side effects (99). Tumor antigens have the capacity to activate immune cells and provoke robust immune responses (100, 101). For instance, vaccination targeting HR-HPV E6 and E7 oncoproteins has demonstrated the ability to induce T-cell responses specific to HPV-16 and even achieve complete histologic responses in certain patients (102, 103). Nevertheless, the presence of immunosuppressive elements within the TME, coupled with its dynamic and diverse attributes, can substantially hinder the clinical efficacy of tumor vaccines in advanced HNSCC (104).

Nanomedicine has achieved considerable advancements, providing a viable methodology to augment the efficacy of vaccines and counteract immunosuppression (105). Initially, nanomaterial-based tumor vaccines can efficiently convey tumor antigens and immune adjuvants to stimulate antigen presentation and amplify the immune response. Secondly, highly immunogenic nano-vaccines can be directly instilled at the tumor site to evoke acquired immunity and eradicate tumor cells. Hydrophilic polyester polymeric nanoparticles constructed of polylactic acid-hydroxymethylglycolic acid (pLHMGA), have been employed in the formulation of therapeutic HR-HPV vaccines. These nanoparticles are laden with a lengthy peptide derived from the HPV16 E7 oncogene and enveloped with toll-like receptor 3 (TLR3) ligand (poly I: C) to augment the immune response. This strategy has demonstrated a significant augmentation in the proportion of HPV-specific CD8^+^ T cells (106). Furthermore, the implementation of E6/E7-oriented nanosatellite vaccines has shown a remarkable augmentation of more than 12-fold in tumor-specific CD8^+^ T cells, culminating in diminished tumor burden (51). Similarly, the application of mesoporous silica rods (MSR)-based HPV-16 E7 vaccines has led to extensive infiltration of antigen-specific CD8^+^ T cells in MOC2-E6E7 tumor cells. This infiltration has been associated with inhibited tumor growth and prolonged survival in MOC2-E6E7 tumor mice, particularly in the HNC models with specific antigen expression (52). Recently, a novel ribonucleic acid lipoplex (RNA-LPX)-based HPV16 vaccine (E7 RNA-LPX) combined with local radiotherapy has been reported to induce a substantial infiltration of E7-specific CD8^+^ T cells in the tumor. This combination therapy has the potential to convert a “cold” tumor into a “hot” tumor (53). Currently, phase I and II clinical trials (NCT04534205, NCT03418480) are underway to investigate the efficacy of the HPV16 E6/E7 RNA-LPX vaccine against various HPV-driven cancers, including HNSCC. Additionally, there are other clinical trials (NCT04287868, NCT04260126, and NCT05232851) that are exploring the therapeutic potential of nano-vaccines in HNSCC.

Moreover, virus-like NPs (VLPs) derived from plants and bacteria have shown high immunogenicity, such as cowpea mosaic virus (CPMV) or tobacco mosaic virus (TMV) (107–109). CPMV nanoparticles are stable, non-toxic, scalable in production, and can be modified with drugs and antigens. Additionally, novel virus-like hollow mesoporous silica nanoparticles (VH-MSNs) have been developed by mimicking the structural characteristics of viruses using an autophagy perovskite template. The distinctive surface topology of VH-MSNs amplifies cellular internalization and magnifies immune responses. These NPs can accommodate doxorubicin, facilitating the integration of chemotherapy and immunotherapy (110). Similarly, in a preclinical model of HNSCC, orthotopic inoculation of virus-like microparticle-coated toll-like receptor 9 agonist (CMP-001) instigated both local and distal antitumor immune responses (111).

#### 3.2.3 Nanomedicines for HNSCC by polarizing TAMs

**Figure 5:**
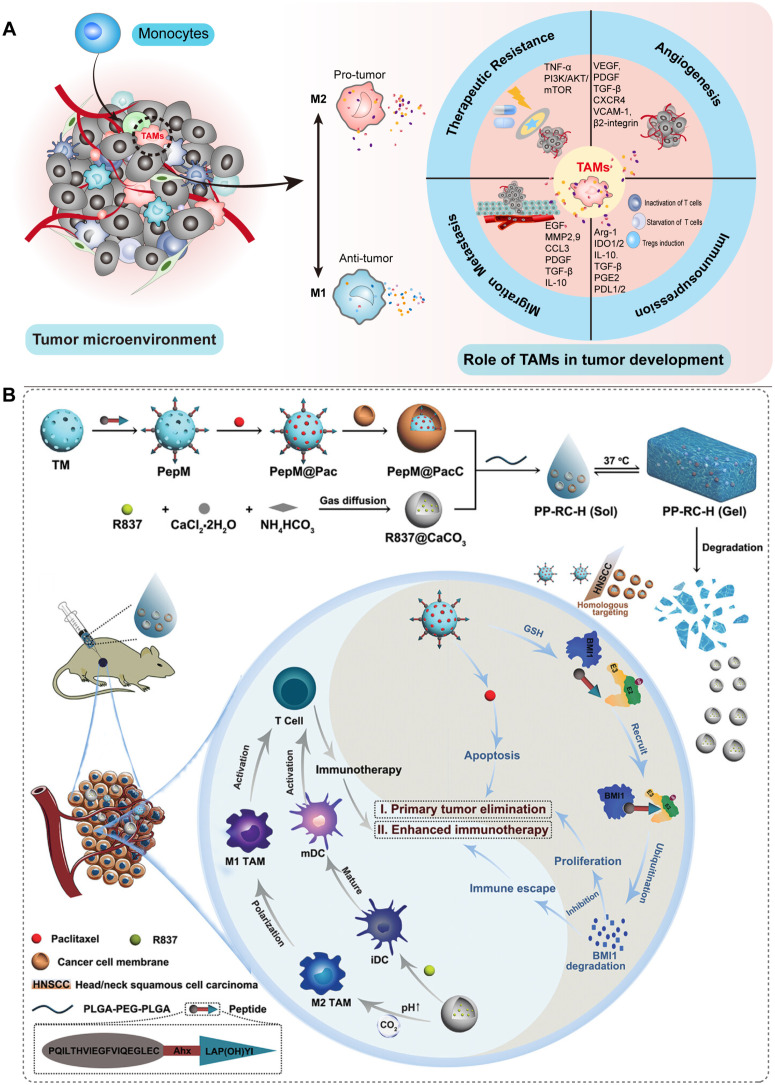
Schematic illustration of the involvement of TAMs in HNSCC and their potential as therapeutic targets for nanomedicine-based treatment of HNSCC. **(A)** TAMs primarily consist of M1 and M2 phenotypes. They play crucial roles in angiogenesis, resistance to treatment, cellular migration, and metastasis, as well as immunosuppression within the TME. **(B)** A nanocomposite hydrogel with antitumor properties for the treatment of primary and metastatic HNSCC (54).

M2 TAMs are known to contribute to tumor progression by fostering tumor growth, angiogenesis, and metastasis (**Figure 5**). They create an immunosuppressive barrier that impedes effective anti-tumor immune responses, thus hampering immune surveillance against the tumor (112). In HNSCC, the predominance of M2 TAMs is notable, and their presence is closely linked to an unsatisfactory overall prognosis (113, 114). Encouragingly, studies have provided compelling evidence showcasing the effectiveness of TAMs-targeting strategies in diverse tumor types (115–118). However, the clinical applicability of current small-molecule drugs targeting TAMs faces several challenges (119).

Nanomaterial-based drug delivery systems have dramatically revolutionized the field of TAM-related immunotherapy (119–123). Rodell et al. synthesized a nanoparticle called CDP-R848, a potent agonist of TLR7 and TLR8. CDP-R848 has been shown to convert TAMs to the M1 phenotype, leading to suppressed tumor growth and prevention of tumor re-invasion in various tumors. Notably, when combined with anti-PD-1 therapy, CDP-R848 has shown an improved response to immunotherapy, even in tumors that were resistant to anti-PD-1 treatment (124). A prodrug formulation called R848-Toco, coupled with α-tocopherol, has been formulated as a polymeric nanosuspension using tocopherol-modified hyaluronic acid (HA-Toco). This delivery system offers prolonged release kinetics and maintains the activity of R848. In the HNC model, subcutaneous injection of the nanosuspension has been shown to recruit immune cells in the TME and achieve significant anti-tumor effects (125). Furthermore, NPs constructed from mRNA encoding interferon regulatory factor 5 and IKKb kinase have demonstrated the ability to reprogram TAMs from the M2 to M1 phenotype. This reprogramming leads to the induction of tumor immunity and anti-tumor effects in ovarian cancer, melanoma, and glioblastoma (126). Recently, there have been reports on the utilization of a nano-platform called M1mDDTF, which involves disguising the iron supply regeneration system with the membrane of M1-type macrophages. This innovative approach has shown promising results in promoting the gradual polarization of TAMs towards the M1 TAMs (127).

The CD47, a signal found on tumor cells that essentially tells immune cells “don’t eat me”, can interact with signal regulatory protein α (SIRPα) on macrophages. This interaction effectively prevents macrophages from engulfing and eliminating the tumor cells. Additionally, tumor cells release certain factors that push macrophages toward the M2 phenotype, further aiding the growth of the tumor. Therefore, Rao et al. developed hybrid membrane nano-vasomes (hNVs) that address both mechanisms simultaneously. These hNVs operate by inhibiting the CD47-SIRPα signaling pathway, thereby facilitating macrophages in recognizing and eliminating tumor cells. Additionally, the hNVs facilitate the repolarization of the M2 to M1 phenotype. This repolarization process helps to inhibit local tumor recurrence and distant metastasis of melanoma (128). The stimulator of interferon genes (STING) is a crucial molecule involved in transmitting signals within the innate immune response (129). Researchers loaded STING agonists into hollow nanovesicles, which resulted in significant suppression of tumor growth in a model of triple-negative breast cancer with low immunogenicity (128).

The lysosomes of TAMs have elevated cysteine protease activity, which hinders antigen cross-presentation and CD8^+^ T cell activation. Cui et al. developed a DNA nanoparticle targeting TAMs lysosomes to specifically inhibit cysteine proteases, improve the ability of TAMs to cross-present antigens, and inhibit tumor growth (130). The latest research reports an injectable nanocomposite hydrogel with a polymer framework (PLGA-PEG-PLGA) loaded with imiquimod-coated CaCO_3_ nanoparticles (RC) and cancer cell membrane (CCM)-coated mesoporous silica nanoparticles PepM@PacC, in which RC not only facilitates the maturation of DCs but also enhances the polarization of TAMs towards the M1 phenotype. Moreover, this nanocomposite hydrogel has demonstrated the ability to improve the TME, suppress tumor growth, and notably impede tumor metastasis to the lungs in a mouse model of HNSCC (54). Despite the use of nanomedicine to target TAMs in HNSCC is still being investigated, it holds significant potential to enhance the therapeutic outcomes of HNSCC. For instance, it has been reported that the combination of intratumoral injection of TLR7 and TLR9 agonists with anti-PD-1 can promote the transformation of TAMs from M2 to M1 and inhibit tumor growth in HNSCC (131). Converting M2 into M1 TAMs using small interfering RNA (siRNA) is a highly promising approach, where the involvement of nanosystems cannot be overlooked (132). Conspicuously, it has been demonstrated that maintaining an appropriate balance between M1 and M2 in the TME (instead of completely polarizing towards M1) can enhance the therapeutic effect mediated by NPs (133).

#### 3.2.4 Nanomedicines treat HNSCC by increasing tumor-infiltrating lymphocytes

The insufficient presence of TILs within the TME significantly hampers the immunotherapy effectiveness. Otherwise, the depletion of T cells is also one of the important factors affecting the effect of immunotherapy (134). Strategies aim to increase tumor-specific TILs in two ways: promoting TIL infiltration in the TME and enhancing APC function to improve T cell activity.

**Figure 6:**
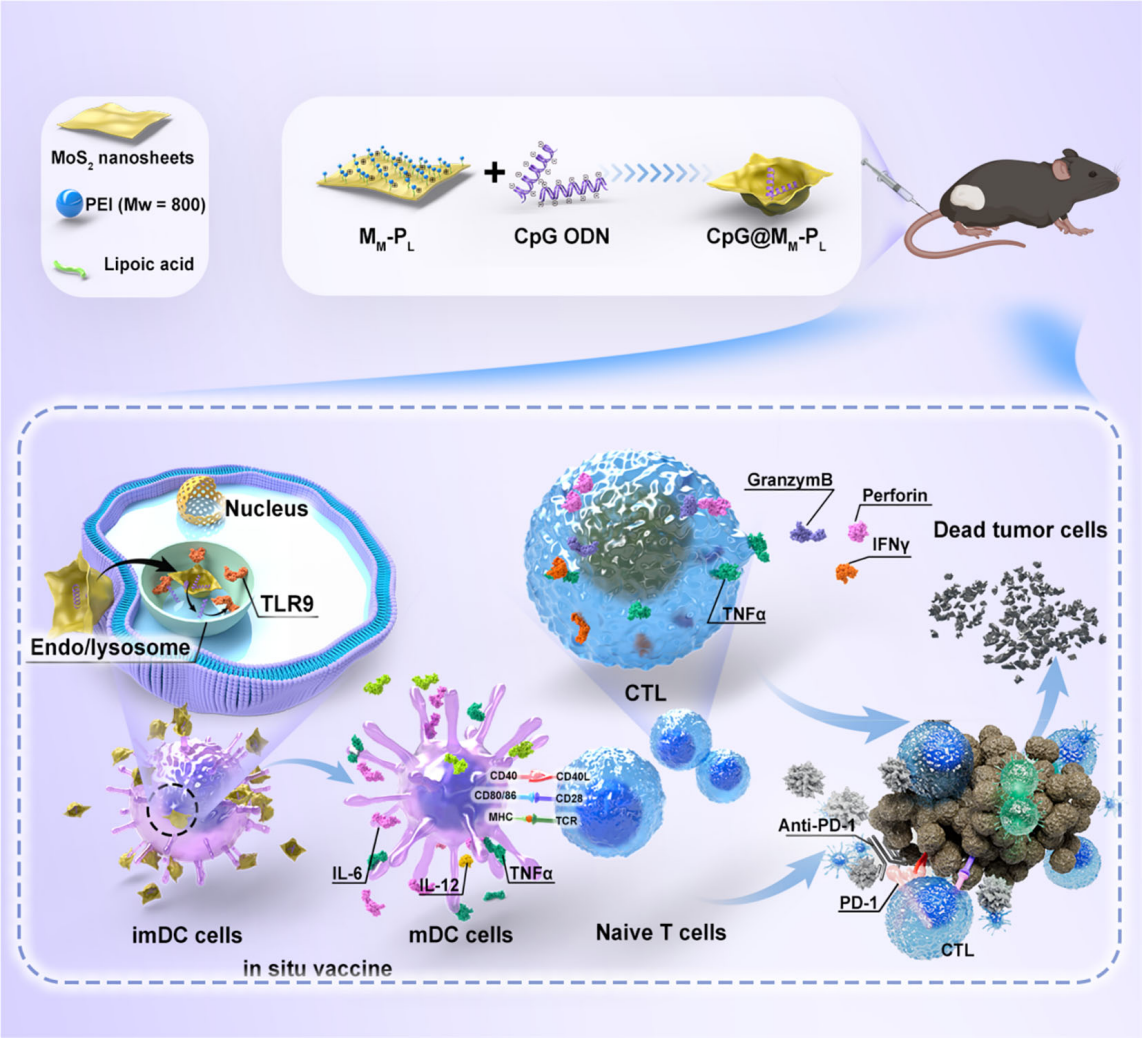
Schematic representation of the antitumor effects of the functionalized nano-platform. The CpG-loaded nanosheets elicit an anti-tumor immune response, enhance the maturation and antigen presentation capacity of BMDCs, and further augment the tumor cytotoxicity of CTLs, thereby ultimately advancing the immunotherapeutic efficacy against HNSCC (56).

Studies have demonstrated the crucial role of the host STING pathway in the production of type I interferon, activation of DCs, and stimulation of CD8^+^ T cells in response to tumor-associated antigens (135, 136). As a result, STING agonists are being developed as a novel therapeutic agent for the treatment of cancer (137). Nonetheless, their effectiveness is limited by barriers to drug delivery. Consequently, Cheng et al. encapsulated the STING agonist cGAMP in liposomes (cGAMP NPs), which markedly improved the cellular uptake of cGAMP, stimulated the secretion of IFN-γ, and increased the infiltration of CD4^+^ and CD8^+^ T cells into the TME. As a result, cGAMP NPs effectively inhibited the growth of triple-negative breast cancer and B16F10 melanoma (138). Furthermore, nanosatellite vectors loaded with STING agonists demonstrated a remarkable 12-fold increase in the infiltration of tumor-specific CD8^+^ T cells in the TME (51). As previously described with nanomedicine PepM@PacC, CaCO_3_ nanoparticles promote DCs maturation and activate T cells, leading to synergistic tumor cell killing and effective inhibition of HNSCC metastasis (54). In certain studies, nanoparticles have been modified by incorporating PD-L1 antibodies to create nanomedicines known as all-trans retinoic acid-polylactic acid-glycolic acid copolymer (PLGA)-PEG-PD-L1. These nanomedicines are designed to specifically target tumor cells. By activating CD8^+^ T cells surrounding PD-L1^+^ tumor cells, the anti-tumor activity is enhanced, effectively converting “cold” tumors into “hot” tumors (55). Importantly, our previous study demonstrated that the functionalized two dimensions nanomaterials MoS_2_ loaded with TLR9 agonist CpG effectively suppressed tumor growth and significantly prolonged mouse survival in the HNSCC model by augmenting TILs within the TME (56) (**Figure 6**).

As mentioned earlier, NPs carrying tumor vaccines can indirectly promote the infiltration of TILs in the TME by activating APCs (53). In addition to improving DCs targeting, the surface of nanoparticles can be modified with antibodies or ligands that selectively bind to the surface receptors of DCs, including C-type lectin receptors, mannose receptors, DCIR2, and CLEC9A (139). Surface modification of NPs can also augment the antigen transport and cross-presentation capabilities of DCs. For instance, NPs modified with polyethylene imine and aluminum hydroxide are more readily endocytosed by DCs, leading to improved release of antigens into the cytoplasm, enhancing antigen cross-presentation ability, and facilitating infiltration of TILs (140).

Recent research has advanced our understanding of the connection between microorganisms and tumor development. It has revealed the complex relationship between commensal bacteria and the host immune system (141, 142). Studies have revealed that lactobacillus, for example, can inhibit the onset of oral squamous cell carcinoma (OSCC) by enhancing the anti-tumor immune response (143), indicating that modulation of the oral microbiota can potentially boost the immune response. Furthermore, Zheng et al. made an intriguing discovery that tumor tissues in OSCC harbored elevated levels of peptostreptococcus bacteria, which were found to be positively associated with survival rates (57). In response, a hydrogel based on silver NPs was developed to suppress the growth of competing bacteria, including peptostreptococcus. The hydrogel was designed to co-deliver anaerobes (which increase the levels of peptostreptococcus) along with exogenous anaerobes and anti-PD-1 therapy. This combination significantly increased the infiltration of CD8^+^ T cells, resulting in inhibited tumor growth in subcutaneous xenograft tumors and 4-NQO-induced spontaneous tongue cancer tumor models (57).

#### 3.2.5 Nanomedicines treat HNSCC by inducing ICD

In recent years, several emerging tumor treatment methods have been discovered, including photodynamic therapy (PDT), photothermal therapy (PTT), and sonodynamic therapy (SDT) (144–146). These therapies utilize photodynamic or sonodynamic approaches to induce death in tumor cells, which release a significant amount of damage-associated molecular patterns (DAMPs), including calretinin, adenosine 3-phosphate (ATP), and high mobility group box 1. DAMPs play a crucial role in activating the adaptive immune response of T cells and promoting the formation of long-term immune memory (147). Consequently, the ICD has the potential to reverse the “cold” TME and enhance the effectiveness of immunotherapy.

Nanomedicine offers great potential in enhancing the efficacy of tumor immunotherapy by inducing ICD of tumor cells, through its high component selectivity and functional modification capabilities (147–150). As an illustration, the photosensitizer mTHPP (5,10,15,20-tetra hydroxyphenyl porphyrin) can be effectively incorporated into polymeric micelles, thereby enhancing the efficiency of PDT (58). As mentioned earlier, M1mDDTF not only possesses the ability to directly eliminate tumor cells but also exhibits the potential to stimulate ICD and facilitate DCs development (127). Additionally, Hackenberg et al. synthesized zinc oxide nanoparticles capable of inducing ICD via photocatalysis, highlighting the potential of PDT as a treatment option for HNSCC (59). Notably, certain proteins such as matrix metalloproteinase (MMP)2 and MMP9, which are overexpressed in the tumor, have shown promise in the enrichment of gelatin nanoparticles, further enhancing their therapeutic potential (151, 152). As a result, researchers have developed gelatin nanoparticles (GENPs) that can be degraded by MMP2 and MMP9. These GENPs were loaded with the photosensitizer indocyanine green (ICG) and the STAT3 inhibitor NSC74859 (NSC, N). Upon near-infrared irradiation, the released ICG can effectively utilize the photothermal effect to kill tumor cells, while NSC can stimulate effective anti-tumor immunity, consequently enhancing the efficacy of tumor therapy (60). Furthermore, Zhou et al. developed a novel nanocomposite called TiO_2_@Ru@siRNA, which involved the modification of TiO_2_ nanoparticles with a ruthenium-based photosensitizer (Ru) and the loading of hypoxia-inducible factor-1α (HIF-1α) siRNA. When exposed to visible light, TiO_2_@Ru@siRNA exhibited both type I and II photodynamic effects. In addition to causing lysosomal damage and silencing the HIF-1α, it was also effective in killing OSCC cells. By alleviating hypoxia and inducing pyroptosis, a form of programmed cell death, TiO_2_@Ru@siRNA can significantly inhibit tumor progression by downregulating key immunosuppressive factors, upregulating immune stimulating factors, and thereby reestablishing the immune microenvironment and enhancing anti-tumor immunity (61). Moreover, TiO_2_@Ru@siRNA-mediated PDT has demonstrated significant efficacy in both xenograft tumor models and rat oral cancer models (61). As mentioned earlier, Au@MC38 can enhance radiosensitivity, moreover, it also elicits local radiation-ICD, which activates a robust immune response and leads to a notable augmentation of CD8a^+^ DCs within the TME (73).

However, the irreversible hypoxic conditions in the TME of HNSCC hinder the effectiveness of PDT. To address this challenge, researchers have focused on chemokinetic approaches by exploring iron-dependent cell death mechanisms with ROS cytotoxicity. Ferroptosis, a non-apoptotic programmed cell death process, not only improves the efficiency of PDT by providing oxygen to the hypoxic TME but also stimulates an immune response through ROS production (153). For instance, a 2-in-1 nanoplatform, known as SRF@Hb-Ce6, loaded with the ferroptosis promoter sorafenib was constructed by linking hemoglobin (Hb) to the photosensitizer chlorinated e6 (Ce6) (154). Furthermore, amphiphilic MMP2-responsive peptides are incorporated into the backbone of the nano-platform to ensure drug release specificity and enhance safety. SRF@Hb-Ce6 has the potential to improve the efficacy of both PDT and ferroptosis. Notably, PDT not only recruits immune cells to secrete IFN-γ, thus promoting the process of ferroptosis but also enhances the sensitivity of tumors to ferroptosis. These findings underscore the promising prospects of combining nanoplatforms with PDT and ferroptosis in cancer treatment (154). Zhu et al. developed a novel supramolecular nanomedicine by self-assembling the photosensitizer Ce6 and ferroptosis-inducing agent erastin through hydrogen bonding and π-π stacking. This nanomedicine, known as Ce6-erastin, exhibits low toxicity to normal tissues but can cause excessive accumulation of ROS, increase the oxygen concentration in tumor cells, and inhibit SLC7A11 expression. Consequently, it enhances toxicity to tongue cancer CAL-27 cells and demonstrates significant anti-tumor activity against OTSCC transplanted tumors after radiation treatment (62).

### 3.3 Other applications of nanomedicines in the treatment of HNSCC

Moreover, nanomaterials are widely utilized as nanocarriers for targeted delivery of chemotherapy drugs, small molecule inhibitors, and nucleic acid agents (155–157). Notably, albumin-bound paclitaxel (NAB-paclitaxel) is an FDA-approved nanomedicine widely used in anti-tumor therapy. Jessica et al. conducted a single-center retrospective analysis, revealing the efficacy of NAB-paclitaxel in the treatment of R/M HNSCC that had shown progression after prior use of other taxanes, such as cremophor-based paclitaxel or docetaxel (158). Furthermore, Wang et al. employed nanomaterial modification techniques to develop NR7 ligand-modified PLGA-PEG/NR7 nanoparticles that could target tumor sites and were simultaneously loaded with cisplatin. In OSCC cells with an overexpression of the receptor, the NR7 peptide could be specifically delivered and rapidly taken up by tumor cells, thereby exerting a potent anti-tumor effect (63).

Targeted delivery of small molecule inhibitors via NPs shown exceptional efficacy in HNSCC treatment. Src, a non-receptor tyrosine kinase, plays a pivotal role in the progression and metastasis of HNSCC. Encapsulating the Src inhibitor saracatinib within NPs has demonstrated significant inhibition of HNSCC metastasis, surpassing the effectiveness of the free drug. Additionally, the co-delivery of the saracatinib (AZD0530) and the AKT inhibitor capivasertib (AZD5363) via cathepsin B-sensitive NPs significantly enhanced the antitumor effect while minimizing side effects. This is primarily attributed to the highly specific and efficient tumor targeting achieved by nanomedicine (64, 65). NAD(P)H: quinone oxidoreductase 1 (NQO1) is an enzyme that is frequently upregulated in squamous cell carcinoma, exerting a significant influence on tumor proliferation and chemotherapy resistance. β-lapachone is a small molecule inhibitor of NQO1. MSN loaded with 5-FU/β-lapachone (FNQ-MSN) can effectively target NQO1, inhibit its expression, and overcome 5-FU resistance, resulting in enhanced cytotoxicity against HNSCC (157). Furthermore, nanoparticles loaded with the novel AKT/PDK1 inhibitor PHT-427 have also demonstrated enhanced anti-tumor effects in HNSCC (66).

siRNA holds great potential as an effective tool for cancer therapy. However, its clinical application faces numerous obstacles, including susceptibility to degradation by ribonucleases, poor stability in physiological conditions, potential to induce inflammatory responses, and lack of site-specificity. Researchers are actively investigating the use of nanoparticles as carriers for efficient siRNA delivery. As an illustration, the use of nanoparticles has demonstrated the ability to suppress the proliferation of HNSCC through the delivery of siRNA targeting ribonucleotide reductase subunit M2. In HNSCC xenografts models, nanoparticles can accumulate in tumors with intravenous injection and the targeted gene knockout effect can persist for 10 days, leading to significant inhibition of tumor progression (159). Additionally, CXCR4 is an attractive target for drug delivery in HNSCC therapy (160, 161). In recent years, DNA tetrahedron-based nanostructures have emerged as promising platforms for siRNA delivery, garnering significant attention (162, 163).

Nanobody (NB) is an innovative immunoglobulin discovered in the serum of Camelidae. It presents the advantages of petite size, potent specificity, elevated stability, facile expression, and the capacity to identify concealed epitopes. It demonstrates a vast scope of application value across various fields and has progressively evolved as a nascent force in the next-generation of therapeutic biomedicals and clinical diagnostic reagents (164). A multiattribute platform consisting of a nanoantibody recognizing the outer membrane domain (EGa1) of EGFR coupled to PEG-liposomes remarkably downregulated EGFR to suppress tumor proliferation both *in vitro* and *in vivo* (165). Nanoantibody-directed photodynamic therapy (NB-PDT) is an efficacious tumor-selective treatment methodology utilizing NBs to specifically transport photosensitizers (PSs) to tumor cells, typically targeting EGFR in conjunction with PDT. Since 2014, NB -directed photodynamic therapy (NB-PDT) has matured into a highly precise tumor treatment method (166, 167). For instance, one investigation characterized two EGFR-directed nanobody-photosensitizer conjugates (NB-PSs), the monovalent 7D12-PS and the bivalent 7D12-9G8-PS, which exhibited significant tumor colocalization (168). In feline oral carcinoma, NB-PDT hasachieved a significant anti-tumor effect (169). Furthermore, amalgamating the photosensitizer IRDye700DX with a nanoantibody targeting EGFR, this nanoantibody-PS conjugate resulted in extensive tumor necrosis (approximately 2/3) with minimal toxicity to healthy tissues in a model of HSNCC (167).

### 3.4 Combination therapy of nanomedicine with other therapeutic approaches

Combining surgery, radiotherapy, chemotherapy, immunotherapy, and targeted therapy is often necessary for effective tumor treatment. However, single-drug therapy has limitations, leading to suboptimal efficacy and side effects. To address this, integrating nanotechnology with various therapeutic approaches shows promise for future research.

Currently, there is increasing research on combining nanomedicine for the treatment of HNSCC. One notable example is that the combination of photocatalytically active zinc oxide subnanoparticles with chemotherapy drugs, cisplatin, and paclitaxel, exerts a significantly stronger effect on the proliferation of HNSCC cells compared to single-drug therapy (170). Furthermore, coordination polymer-based core-shell NPs loaded with cisplatin and photosensitizer pyrolipid (NCP@pyrolipid) have demonstrated the ability to induce apoptosis and necrosis of cancer cells synergistically. In a cisplatin-resistant HNC SQ20B xenograft model, NCP@pyrolipid exhibited remarkable tumor regression (83% reduction in tumor volume) at low doses (171). As mentioned, the ICD in combination with other treatments to stimulate the anti-tumor immune response may be one of the strategies for combination therapy (60, 61). Additionally, numerous studies have explored combination therapy approaches concerning nanotechnology, including nano-vaccine therapy combined with local radiotherapy (53), the delivery of siRNA or gene editing through nanomaterials combined with chemotherapy (172–174), and PTT combined with chemotherapy (175, 176).

## 4 Summary and prospects

Nanotechnology has significantly advanced therapeutic drug delivery in cancer treatment. Several nanomedicines, encompassing Doxil (1995), Feraheme (2009), and albumin-bound paclitaxel nanoparticles (Abraxane, 2005), have surfaced and are presently employed in clinical applications (177). These modalities have widened the therapeutic efficacy for HNSCC. Numerous clinical studies have explored the conceivable utility of nanomedicine for diagnosing and treating HNSCC (**Table 2**). For example, the Abraxane combination therapy has exhibited promising outcomes in the context of N2b lymph node invasion or unresectable HNSCC (178). A single-arm, multi-center, phase II trial indicated superior objective response rate and OS with albumin-bound paclitaxel, cetuximab, and carboplatin (CACTUX) as compared to the EXTREME regimen in patients afflicted with R/M HNSCC (179). In 2021, NBTXR3 nanoparticles were employed as a radiosensitizer for the management of locally advanced OSCC patients eligible for radiotherapy. Phase I clinical trials confirmed its safety, paving the way for ongoing phase II trials (180). Notably, phase II/III clinical trials (NCT04892173 and NCT04862455) are presently enlisting participants to evaluate the therapeutic efficacy of NBTXR3 nanoparticles in locally advanced and R/M HNSCC. Furthermore, multi-center, phase II-III randomized controlled trials have validated the efficacy of NBTXR3 in soft tissue sarcomas (181). Additionally, mRNA-2752, a lipid nanoparticle encapsulating mRNAs encoding human OX40L, IL-23, and IL-36γ, is currently undergoing clinical trials for various malignancies, including HNSCC (NCT03739931).

**Table 2:** Summary of clinical trials of nanomedicine for HNSCC treatment.

Nanotechnology platform	Intervention/Treatment	Disease	Phase	NCT #
Functionalized HfO_2_ NPs	NBTXR3/RT ± Cetuximab	LA-HNSCC	III	NCT04892173
Functionalized HfO_2_ NPs	NBTXR3 ± Pembrolizumab	R/M HNSCC	II	NCT04862455
Nab-paclitaxel	Abraxane+Cetuximab + IMRT	Stage III-IV HNSCC	I	NCT00736619
Nab-paclitaxel	Nab-paclitaxel+Cisplatin+5-FU	LA HNSCC	II	NCT01566435
Nab-paclitaxel	Nivolumab+Nab-paclitaxel+Carboplatin	HPV+ HNSCC	II	NCT03107182
Oxide NPs	Ferumoxytol+MRI	LA-HNSCC	I	NCT01895829
Lipid NPs	mRNA-2752 ± ICIs	R/R solid tumor or lymphoma	I	NCT03739931
Docetaxel NPs	BIND-014	HNSCC	II	NCT02479178
Nab-paclitaxel	Nab-paclitaxel+CRT	Recurrent HNSCC	I	NCT01847326
Nab-paclitaxel	EphB4-HSA ± Nab-paclitaxel	Advanced/Metastatic Tumors	Ib	NCT02495896
Functionalized HfO_2_ NPs	NBTXR3+Pembrolizumab	Recurrent HNSCC	II	NCT04834349
Quercetin Encapsulated NPs	Quercetin Encapsulated NPs	TSCC	II	NCT05456022
Nab-paclitaxel	Nab-paclitaxel, Cisplatin+Cetuximab+RT	LA-HNSCC	I/II	NCT00851877
Polymeric Micelles	NC-6004 + 5-FU+Cetuximab	HNC	I	NCT02817113
Nab-paclitaxel	Nab-paclitaxel+Carboplatin	Stage III/IV HPV+ OSCC	II	NCT02258659
Nab-paclitaxel	Nab-Paclitaxel ± Cisplatin	LA-HNSCC	II	NCT02573493
Functionalized HfO_2_ NPs	NBTXR3+anti-PD-1	LR or R/M HNSCC	I	NCT03589339
Nab	Nab Rapamycin	Advanced mTOR-mutated Tumors	I	NCT02646319
Polymeric Micelles	NC-6004 + 5-FU+Cetuximab	R/M-HNSCC	I/II	NCT03109158
Polymeric Micellar	Genexol-PM+Cisplatin	LA-HNSCC	II	NCT01689194
Polymeric Micelle	Docetaxel-PM	R/M-HNSCC	II	NCT02639858
Lipid NPs	PDS0101+M7824+NHS-IL-12	HPV+ malignancies	I/II	NCT04287868

HfO_2_, hafnium oxide; Nab, Nanoparticle albumin-bound; LA HNSCC, Locally Advanced HNSCC; LR, Locoregional Recurrence; R/R, Relapsed/Refractory; TSCC, Tongue Squamous cell carcinoma; IMRT, Intensity Modulated Radiotherapy; RT, Radiation Therapy; CRT, Chemoradiotherapy.

Nevertheless, several obstacles need to be surmounted before translating preliminary nanomedicine research into triumphant clinical applications. A pivotal challenge is achieving equilibrium between the intricacy of nanomedicine constituents, manufacturing expenses, and therapeutic potency (182). The uniformity and stability of nanomedicine batches are equally crucial for enduring clinical advantages. Additionally, apprehensions concerning pharmacokinetics, efficacy, and long-term safety require meticulous scrutiny. Discrepancies between animal models and human physiology may affect the dependability of preliminary study outcomes. Tumor heterogeneity and the necessity for patient stratification further impact the effectiveness of nanomedicines. Identifying credible biomarkers or characteristics for patient selection in HNSCC nanomedicine treatments necessitates further research.

Preserving a positive perspective on the potential potency of nanomedicine in treating HNSCC is paramount. Preclinical studies have exhibited extraordinary effects and hold substantial promise for the discipline. Addressing the obstacles outlined above necessitates collaborative endeavors across disciplines like materials science, nanoscience, chemistry, biology, medicine, and pharmacy. By nurturing interdisciplinary collaborations, we can strive towards the successful clinical implementation of nanomedicines, ultimately improving the prognosis and results for cancer patients.
